# Gestational diabetes is associated with the risk of offspring’s congenital anomalies: a register-based cohort study

**DOI:** 10.1186/s12884-023-05996-6

**Published:** 2023-10-03

**Authors:** Jenni Kinnunen, Hilkka Nikkinen, Elina Keikkala, Sanna Mustaniemi, Mika Gissler, Hannele Laivuori, Johan G. Eriksson, Risto Kaaja, Anneli Pouta, Eero Kajantie, Marja Vääräsmäki

**Affiliations:** 1 Wellbeing Services County of North Ostrobothnia, Department of Obstetrics and Gynecology, Research Unit of Clinical Medicine, Medical Research Center, Oulu University Hospital, University of Oulu, Oulu, 90029 Finland; 2 Finnish Institute for Health and Welfare, Population Health, Public Health and Welfare, Helsinki, Oulu, 00271, 90101 Finland; 3 Department of Knowledge Brokers, Finnish Institute for Health and Welfare, Helsinki, 00271 Finland; 4 Academic Primary Health Care Centre, Region Stockholm, Department of Molecular Medicine and Surgery, Karolinska Institute, Stockholm, 17176 Sweden; 5 Department of Obstetrics and Gynecology, Faculty of Medicine and Health Technology Tampere University Hospital, Tampere University, Tampere, 33100 Finland; 6 Medical and Clinical Genetics, Helsinki University Hospital, University of Helsinki, Helsinki, 00014 Finland; 7 Institute for Molecular Medicine Finland, Helsinki Institute of Life Science, University of Helsinki, Helsinki, 00014 Finland; 8 Department of General Practice and Primary Health Care, Helsinki University Hospital, University of Helsinki, Helsinki, 00014 Finland; 9 Folkhälsan Research Center, Helsinki, 00250 Finland; 10 Department of Obstetrics and Gynecology, Human Potential Translational Research Programme, Yong Loo Lin School of Medicine, National University of Singapore, Singapore, 119228 Singapore; 11 Turku University Hospital, Turku University, Turku, 20521 Finland; 12 Department of Government Services, Finnish Institute for Health and Welfare, Helsinki, 00271 Finland; 13 University of Helsinki and Helsinki University Hospital, Children’s Hospital, Helsinki, 00290 Finland; 14 Department of Clinical and Molecular Medicine, Norwegian University of Science and Technology, Trondheim, NO-7491 Norway

**Keywords:** Gestational diabetes, GDM, Pregnancy outcome, Offspring, Major congenital anomalies, Chromosomal abnormalities, Aneuploidy, Non-chromosomal anomalies

## Abstract

**Background:**

Gestational diabetes mellitus (GDM) is a common pregnancy-related disorder and a well-known risk factor for adverse pregnancy outcomes. There are conflicting findings on the association of GDM with the risk of congenital anomalies (CAs) in offspring. In this study, we aimed to determine study whether maternal GDM is associated with an increased risk of major CAs in offspring.

**Methods:**

This Finnish Gestational Diabetes (FinnGeDi) register-based study included 6,597 women with singleton pregnancies and a diagnosis of GDM and 51,981 singleton controls with no diabetes identified from the Finnish Medical Birth Register (MBR) in 2009. Data from MBR were combined in this study with the Register of Congenital Malformations, which includes the data of CAs. We used logistic regression to calculate odds ratios (OR) for CAs, together with their 95% confidence intervals (CIs), adjusting for maternal age, parity, pre-pregnancy body mass index (BMI), and maternal smoking status.

**Results:**

The risk of major CAs was higher in the GDM-exposed (*n* = 336, 5.09%) than in the non-exposed group (*n* = 2,255, 4.33%) (OR: 1.18, 95% CI: 1.05–1.33, *p* = 0.005). The adjusted OR (aOR) was 1.14 (95% CI: 1.00-1.30, *p* = 0.047). There was a higher overall prevalence of CAs, particularly chromosomal abnormalities (0.52% vs. 0.21%), in the GDM-exposed group (OR: 2.49, 95% Cl: 1.69–3.66, *p* < 0.001). The aOR was 1.93 (95% Cl: 1.25–2.99, *p* = 0.003).

**Conclusions:**

Offspring exposed to GDM have a higher prevalence of major CAs. Of note, risk factors other than GDM, such as older maternal age and a higher pre-pregnancy BMI, diminished the between group differences in the prevalence of major CAs. Nevertheless, our findings suggest that offspring exposed to maternal GDM are more likely to be diagnosed with a chromosomal abnormality, independent of maternal age, parity, pre-pregnancy BMI, and smoking.

## Background

Gestational diabetes mellitus (GDM) is defined as a glucose intolerance diagnosed for the first time during pregnancy that does not fulfil the criteria for type 1 or 2 diabetes [1]. Its prevalence is globally increasing along with an increase in childbearing age and obesity among women, with GDM affecting 10–32% of the pregnant population [2]. This wide variation in the incidence of GDM is explained by screening policies and diagnostic criteria applied [2].

GDM has many well-documented short- and long-term consequences for the offspring. These include macrosomia, problems in adaptation to the extrauterine life, and subsequent disturbances including e.g. metabolic and various neurodevelopmental problems [3–5]. One debated outcome of GDM is the risk of congenital anomalies (CAs) among offspring exposed to maternal GDM. A congenital anomaly is defined as structural microscopic or macroscopic variation in the phenotype, with a substantial departure from the reference population during the prenatal period [6]. It is referred to as major when it has or is likely to have significant consequences for an individual’s health. CAs can develop at different times, from the period of genetic recombination during gamete development to the embryonic period with fetal organogenesis and the later gestational period [6, 7]. Studies have reported a link between pre-pregnancy diabetes, particularly maternal hyperglycemia, and CAs in offspring [8, 9]. In GDM-exposed pregnancies, hyperglycemia manifests later in pregnancy as a result of insufficient adaption to pregnancy-related metabolic challenges [10].

Previous studies examining the link between GDM and CAs in offspring have reported conflicting results, with the largest of recent studies and meta-analyses finding that the overall risk of CAs was increased [11–15]. Moore et al. reported that exposure to GDM doubled the risk of chromosomal abnormalities in offspring due to a seven-fold increase in sex chromosomal abnormalities [16]. In this study, our aim was to investigate whether offspring exposed to maternal GDM are more likely to be diagnosed with CAs. To shed light on this, we used data from a large population-based cohort with comprehensive GDM screening.

## Materials and methods

**Fig. 1:**
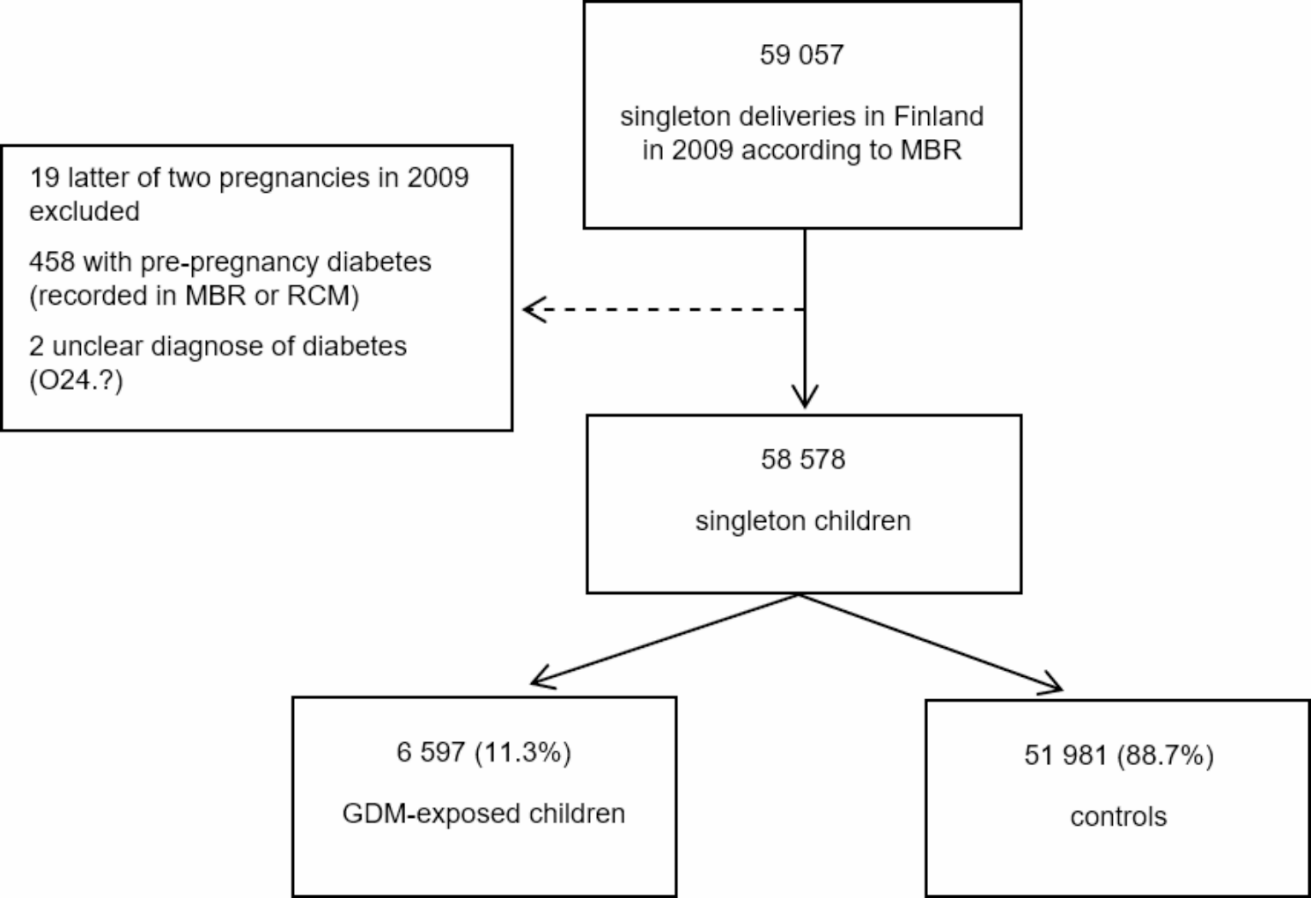
Flowchart of the study population. MBR = Medical Birth Register, RCM = Register on Congenital Malformations; GDM = Gestational Diabetes Mellitus

The Finnish Gestational Diabetes Study (FinnGeDi) has previously been described in greater detail [17]. All singleton pregnancies in Finland in 2009 included in this cohort study were identified from the Finnish Medical Birth Register (MBR). Pregnancies with maternal type 1 or type 2 diabetes were excluded (International Statistical Classification of Diseases and Related Health Problems, 10th Revision [ICD-10] E10, E11, E13, E14.9 and O24.0 -O24.3). If a woman had two deliveries during 2009, the latter was excluded. In total, 58,578 singleton pregnancies (mother-child pairs) were included in the present study. The flow chart of the study population presented in the Fig. 1.

The MBR has been a statutory register since 1987 and is maintained by the Finnish Institute for Health and Welfare (THL). It contains information on all livebirths and stillbirths with gestational age of ≥ 22 + 0 weeks or weighing ≥ 500 g in Finland, with information on maternal background factors, such as age, parity, pre-pregnancy body mass index (BMI), smoking, and pregnancy-related diagnoses (ICD-10), in addition to the main outcomes of pregnancy and the neonatal period in the first seven days. Pregnancy terminations due to malformations or other reasons were not included in this cohort study.

Data on CAs and the subtypes of them among offspring were retrieved from the Register of Congenital Malformations (RCM). The RCM has been a statutory register since 1963 and is currently maintained by the THL. The RCM includes information on live births and stillbirths and CAs and their diagnosis detected prenatally or until the age of one year, as well as information on a few other congenital conditions, such as hypothyroidism. In addition, it contains information on pregnancy-related issues and background information on the mothers. Malformations in the register were classified according to the International Statistical Classification of Diseases and Related Health Problems, 9th Revision (ICD-9) until 2014. A generally approved definition of CA is a structural variation in the phenotype, with a substantial departure from the reference population during the prenatal period, where major CAs also have remarkable consequences for the individual [6]. The criteria for minor congenital malformations and other inconsequential conditions are principally the same as those in the European Concerted Action on Congenital Anomalies and Twins (EUROCAT) guidelines. The focus of the current study is on major CAs. The data in the RCM are compiled from several national healthcare registers and sources. The data are continuously updated, although most of the information is collected during the first two years after birth [18, 19]. The quality and coverage of both the MBR and RCM are considered to be high [20, 21]. These registers were compiled for the study by using personal identification numbers of mothers and their offspring. These identification numbers were pseudonymised by an individual not involved in the study.

Since 2010, all pregnant women in Finland have been offered screening for aneuploidy and congenital malformations, including both combined first-trimester screening with an ultrasound scan at 11–13 + 6 weeks and serum screening at 9 + 0 to 11 + 6 weeks and an ultrasound scan for severe structural abnormalities at 18 + 0 to 21 + 6 weeks. Combined first-trimester screening was offered to 58–87% and second-trimester structural screening to 77–88% of all pregnant women in 2007–2009 [22].

Maternal GDM was identified from the MBR using the following criteria: an abnormal oral glucose tolerance test (OGTT) performed during pregnancy (yes/no), insulin treatment started during the pregnancy (yes/no), and/or a diagnosis of GDM (diagnosis codes O24.4 or O24.9). The sensitivity and specificity of the MBR in the detection of GDM has been validated previously by our group [17]. The accuracy of a GDM diagnosis was 95% [17]. A maternal diagnosis of GDM was accepted also from the RCM. The final study included 6,597 offspring of mothers with GDM and 51,981 offspring of mothers without GDM.

Comprehensive GDM screening of the cohort was performed according to Finnish Current Care Guidelines (FCCG), which recommend a 2-hour 75 g OGTT at 24–28 gestational weeks for all pregnant women, except women of normal weight (BMI: < 25.0 kg/m^2^), primiparous women younger than 25 years and normal weight, and multiparous women younger than 40 years of age with no risk factors for GDM. The cut-off concentrations for venous plasma glucose were as follows: ≥ 5.3 mmol/L fasting and ≥ 10.0 mmol/L 1 h after a glucose load and/or ≥ 8.6 mmol/L 2 h after a glucose load [23]. High-risk factors according to FCCG in 2009 were a history of GDM or fetal macrosomia in a previous pregnancy, obesity with BMI ≥ 35.0 kg/m^2^,glucosuria, a family history of type 2 diabetes, oral corticosteroid medication, or a diagnosis of polycystic ovary syndrome. In the latter cases, OGTT was conducted at 12–16 weeks and if negative, re-testing took place at 24–28 gestational weeks. OGTT testing was also recommended in the case of clinical suspicion of diabetes at any gestational week [23].

Maternal age was defined as age at the time of delivery. Maternal height and pre-pregnancy weight were recorded at the first antenatal visit, and maternal pre-pregnancy BMI was calculated using this information. Maternal smoking status (yes/no) was recorded at the first antenatal visit. Preterm delivery was defined as delivery before 37 + 0 weeks, and perinatal mortality was defined as fetal death at 22 + 0 gestational weeks or later or death in the first week of life. Major CAs are classified principally in the guidelines of EUROCAT. Chromosomal abnormalities were defined as 758.0-758.3 or 758.5-758.9, trisomies as 758.0-758.2 (partial trisomies not included) and sex chromosomal abnormalities as 758.6-758.8 according to the ICD-9 classification.

The FinnGeDi study protocol was approved by the Regional Ethics Committee in Northern Ostrobothnia Hospital District and the THL. Permission for access to the registry data used in the current study was provided by the registry administrator of the THL.

### Statistical analysis

Statistical analyses were performed using IBM SPSS Statistics 27.0 (IBM SPSS Statistics for Windows, Version 27.0. Armonk, NY: IBM Corp). Differences between the GDM and control groups were analysed using a two-sample *t*-test for continuous variables and reported as means with standard deviations (SDs). A χ^2^-test was used to calculate the differences in frequencies in case of categorical variables. Risk estimates for categorical outcomes were calculated using logistic regression analysis and reported as odds ratios (ORs) with their 95% confidence intervals (CIs). The threshold for statistical significance was a *p*-value (*p*) of < 0.05. The data were adjusted for confounding factors, which comprised maternal age at delivery (linear), pre-pregnancy BMI categorized into five groups: <18.5 kg/m^2^, 18.5–24.9 kg/m^2^ (reference group), 25-29.9 kg/m^2^, 30–34.9 kg/m^2^ and ≥ 35 kg/m^2^, parity (linear), and maternal smoking status during pregnancy (yes/no).

To ensure that the nonlinear effect of maternal age to the CAs did not confound the results, we used three different adjustment models where maternal age was: linear (model 1), linear along with quadratic (age^2^) (model 2) and dummy coded (model 3). For dummy coded model maternal age was categorized into seven groups: <20 years, 20–24 years, 25–29 years (control group), 30–34 years, 35–39 years, 40–44 years, and ≥ 45 years.

## Results

The prevalence of GDM was 11.3% in the study population. Mothers diagnosed with GDM were older, more often multiparous, obese, and more likely to smoke as compared to the controls. Gestational age at delivery was slightly lower, and preterm deliveries were more common in GDM pregnancies, but there was no difference in perinatal mortality between the groups (Table 1).

**Table 1:** Characteristics of the mothers and pregnancies

Characteristics	GDM (*n* = 6,597) *n* (%)/mean (SD)	Controls (*n* = 51,981) *n* (%)/mean (SD)	*p*-value
Age at delivery			
Years, mean (SD)	31.1 (± 5.6)	29.3 (± 5.3)	< 0.001*
< 20 20–24 25–29 30–34 35–40 40–44 ≥ 45	107 (1.6%) 700 (10.6%) 1,800 (27.3%) 2,124 (32.2%) 1,345 (20.4%) 469 (7.5%) 25 (0.4%)	1,288 (2.5%) 8,548 (16.4%) 16,917 (32.5%) 16,732 (32.2%) 6,888 (13.3%) 1,556 (3.0%) 52 (0.1%)	
Pre-pregnancy BMI			
kg/m^2^, mean (SD)	28.4 (± 6.1)	23.7 (± 4.3)	< 0.001*
< 18.5 18.5–24.9 25–30 30–34.9 ≥ 35 Missing	84 (1.3%) 2,001 (30.3%) 2,086 (31.6%) 1,389 (21.1%) 889 (13.5%) 148 (2.2%)	2016 (3.9%) 33,781 (65.0%) 10,222 (19.7%) 3,105 (6.0%) 1,213 (2.3%) 1644 (3.2%)	
Parity			
Mean (SD)	1.25 (± 1.6)	1.02 (± 1.4)	< 0.001*
First Second or more	2,394 (36.3%) 4,203 (63.7%)	22,363 (43.0%) 29,618 (57.0%)	
Smoking			< 0.001**
Yes No Missing	1,177 (17.8%) 5,278 (80.0%) 142 (2.2%)	7,802 (15.0%) 42,816 (82.4%) 1,363 (2.6%)	
Gestational age at delivery			
Weeks, mean (SD)	39.6 (± 1.7)	39.9 (± 1.8)	< 0.001*
Preterm birth < 37 weeks Term birth ≥ 37 weeks Missing	333 (5.1%) 6,258 (94.9%) 6 (0.1%)	2,145 (4.1%) 51,095 (95.7%) 76 (0.1%)	
Perinatal mortality			0.436**
Yes No	25 (0.4%) 6572 (96.6%)	232 (0.4%) 51,749 (96.6%)	

*t-test, ** χ2-test, GDM = gestational diabetes mellitus, SD = standard deviation, BMI = body mass index

**Fig. 2:**
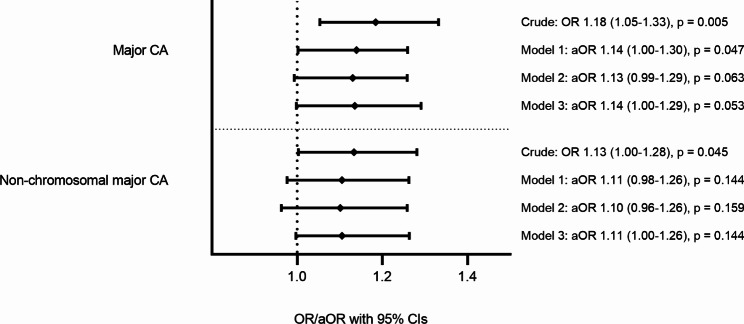
Odds ratios (OR) and adjusted odds ratios (aOR) for major CAs and non-chromosomal major CAs in GDM-exposed offspring. Adjustments: model 1 linear maternal age, categorized pre-pregnancy BMI, parity and smoking status; model 2 linear maternal age, quardratic maternal age, categorized pre-pregnancy BMI, parity and smoking status; model 3 categorized maternal age, categorized pre-pregnancy BMI, parity and smoking status

The risk of major CAs was higher among the offspring of GDM mothers (*n* = 336, 5.09%) compared to the controls (*n* = 2,269, 4.33%) (OR: 1.18, 95% CI: 1.05–1.33, *p* = 0.005). The risk was slightly attenuated after adjustment for maternal age, parity, pre-pregnancy BMI, and maternal smoking (model 1 aOR: 1.14, 95% CI: 1.00-1.30, *p* = 0.047; model 2 aOR 1.13, 95% CI 0.99–1.29, p = 0.063 and model 3 aOR 1.14, 95% CI 1.00-1.29, p = 0.053) (Table 2; Fig. 2).

**Fig. 3:**
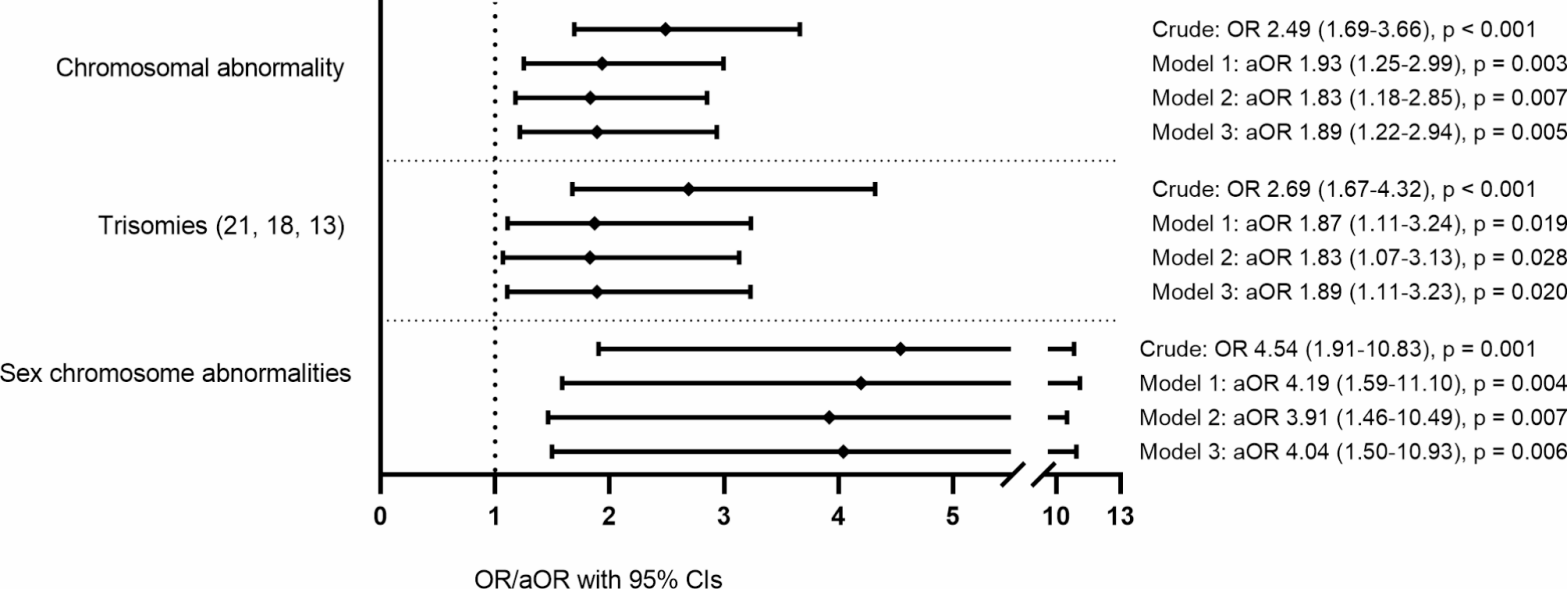
Odds ratios (OR) and adjusted odds ratios (aOR) for all chromosomal abnormalities, trisomies and sex chromosomal abnormalities in GDM-exposed offspring. Adjustments: model 1 linear maternal age, categorized pre-pregnancy BMI, parity and smoking status; model 2 linear maternal age, quardratic maternal age, categorized pre-pregnancy BMI, parity and smoking status; model 3 categorized maternal age, categorized pre-pregnancy BMI, parity and smoking status

Chromosomal abnormalities were more common among offspring of the GDM mothers (*n* = 34, 52/10,000) than among those of the controls (*n* = 108, 21/10,000) (OR: 2.49, 95% Cl: 1.69–3.66, *p* < 0.001), and the increased risk remained after adjustments (model 1 aOR: 1.93, 95% Cl: 1.25–2.99, *p* = 0.003). Furthermore, the proportion of the most common trisomies (trisomy 21, 13, and 18) was higher among the offspring of the mothers with GDM (*n* = 23, 35/10,000) compared to those of the controls (*n* = 68, 13/10,000) (OR: 2.69, 95%, CI: 1.67–4.32, *p* < 0.001; model 1 aOR: 1.87, 95% CI: 1.11–3.24, *p* = 0.019), as well as the proportion of sex chromosome abnormalities (*n* = 8, 12/10,000 and *n* = 14, 3/10,000, respectively) (OR: 4.54, 95% CI: 1.91–10.83; model 1 aOR: 4.19, 95% CI: 1.59–11.10, *p* = 0.004). Changes in the adjustment models had no impact on the results (Table 2; Fig. 3).

We conducted a further subanalysis, including only major CAs without chromosomal abnormalities. The risk of non-chromosomal major CAs remained higher among the offspring exposed to GDM than among the controls (4.7% GDM vs. 4.1%, OR: 1.13, 95% Cl: 1.00-1.28, *p* = 0.045), but this risk was attenuated after further adjustment (model 1 aOR: 1.11, 95% Cl: 0.98–1.26, *p* = 0.144). These analyses were verified by using all the three adjustment models with no impact on the results (Fig. 2).

**Table 2:** Prevalences of different types of abnormalities among the offspring with and without GDM-exposure.

Type of anomalia	GDM-exposed offspring (*n* = 6,597) *n* (%)	Controls (*n* = 51,981) *n* (%)	p-value*
Major CA	336 (5.09%)	2,255 (4.33%)	0.005**
Non-chromosomal major CA	307 (4.65%)	2,153 (4.14%)	0.045**
Chromosomal abnormality	34 (0.52%)	108 (0.21%)	< 0.001**
Trisomies (21, 18, 13)	23 (0.35%)	68 (0.13%)	< 0.001**
Sex chromosomal abnormality	8 (0.12%)	14 (0.03%)	0.001**

GDM = gestational diabetes mellitus, CA = congenital anomalia, *χ2-test, **Statistically significant difference

## Discussion

In our large, population-based cohort comprehensively screened for GDM, we found that the overall prevalence of CAs was 1.1 to 1.2-fold among the offspring exposed to maternal GDM compared to the non-exposed offspring. GDM associated with the risk of CAs although maternal age, pre-pregnancy BMI, parity and maternal smoking status partly explained the risk. Our results are in line with those of the latest meta-analyses, in which GDM-exposure was associated with an increased risk of CAs in offspring [11, 12]. Furthermore, we found chromosomal abnormalities to be more common among the offspring of the mothers with GDM than among the offspring of the controls.

As previously reported, the link between maternal hyperglycemia and an elevated risk of major CAs among offspring is well documented in pre-pregnancy diabetes studies, with hyperglycemia considered a major teratogen [6, 7]. However, other possible mechanisms underlying this link remains uncertain [8, 9]. In addition to hyperglycemia, elevated levels of reactive oxygen species may play a role in increasing the risk of CAs in offspring in diabetes affected pregnancies [6, 13]. Hyperglycemia exists both in pre-pregnancy diabetes and GDM, but in GDM it progresses to fully-fledged GDM or is diagnosed usually not until in the second trimester. Insulin resistance and beta cell proliferation are normal metabolic adaptations during pregnancy [10]. However, pre-existing insulin resistance is common among women with GDM, which increases the risk of insufficient beta cell proliferation, leading to hyperglycemia during pregnancy [10, 24, 25]. Previous research has shown that offspring exposed to GDM are more likely to be diagnosed with major CAs [26]. In Finland, the FCCG has recommended comprehensive GDM screening since 2008. Comprehensive screening will identify more GDM cases with mild hyperglycemia than risk factor-based GDM screening will [27, 28]. While mothers with pre-pregnancy diabetes were excluded in our study, it is unlikely that undiagnosed pre-pregnancy diabetes explains the higher incidence of major CAs or chromosomal abnormalities among the offspring exposed to GDM in our study. This is because the prevalence of undiagnosed pre-existing diabetes is considered to be low [26] and also cases with mild hyperglycemia are identified by using comprehensive screening of GDM [28].

Advanced maternal age increases offspring risk of major CAs in general, and especially CAs derived from autosomal chromosomal abnormalities. However, the risk of non-chromosomal CAs seems not to increase by maternal age [29–33]. Maternal age may increase the risk of chromosomal abnormalities and CAs via many different mechanisms, including telomere shortening affecting genetic recombination in meiosis and an elevated risk of teratogen exposure over time [33]. In our study, advanced maternal age was associated with the risk of both major CAs and chromosomal abnormalities. GDM exposure was still independently linked with a risk of chromosomal abnormalities in offspring.

In a study by Moore et al. (2002) the study population underwent amniocentesis, and GDM was associated with an increased risk of chromosomal abnormalities, specifically sex chromosomal abnormalities. Our study compliments these findings using population-based data on women and comprehensive screening of GDM. We observed chromosomal abnormalities to be more common among offspring of mothers with GDM than those of the controls, with ORs in the order of magnitude of 2 for trisomies and 3 for sex chromosome aneuploidies after adjusting for confounders. Non-chromosomal CAs are considered to occur during the embryonic phase and organogenesis before the 12th gestational week (45). In contrast, chromosomal abnormalities originate at the time of meiosis due to aberrant genetic recombination [34]. The origin of different chromosomal abnormalities varies, but maternal origin dominates in autosomal aneuploidies [34, 35]. For instance, in the case of the most common chromosomal abnormality, trisomy 21, 95% of meiotic stage aberrations are found to be of maternal origin, which is also the case in most autosomal trisomies [7]. In addition, most trisomy 21 cases are constitutional, with only 5% of cases found to be postzygotic [7]. In cases of sex chromosomal abnormalities, maternal and paternal origins are more equal [34–36]. Since GDM is typically diagnosed in the second trimester, GDM should not be considered as a direct risk factor for CAs or chromosomal abnormalities but rather as a signal of a longer-term metabolic dysfunction of the mother, which affects at the time of meiosis and early pregnancy. In this study the total prevalence of major CAs and chromosomal abnormalities differed slightly in the GDM-exposed versus the non-exposed offspring and the prevalence of GDM was reprensative to the nowadays global prevalence of GDM. Major CAs and chromosomal abnormalities cause high morbidity and stress to the family, making the elevated risk of major CAs and chromosomal abnormalities in GDM-exposed offspring an important health concern.

Our study has several strengths. The large study cohort was population based, and the national guidelines for screening and diagnosing GDM were universal [23]. Additional strengths of the present study are that the follow-up of GDM is well-organized at the national level and that screening of fetal abnormalities is comprehensive in Finland. Furthermore, we adjusted the results for the most important confounders, such as maternal age, BMI, parity, and smoking. In Finland ethnicity was relatively homogenous in 2009 [37] and therefore the effect of ethnicity cannot be studied although it has a role as a risk factor of GDM. Finally, the national registers in Finland that we used are inclusive and validated [38].

There are also some limitations. Due to the register setting, there are some factors reported to be linked to the risk of CAs in offspring that we were not able to control for, such as alcohol intake [39]. As comprehensive screening of GDM was not established until 2008, the OGTT was performed in only 43.2% of the study population. This has implications for the data, while some GDM cases might potentially be included among the controls, although such cases would be expected to have a milder form of GDM [40]. The prevalence of GDM in this study was representative concerning the global GDM prevalence, but GDM has still become more common in Finland (which is partially due to comprehensive screening procedure becoming more familiar). In 2019 the prevalence of GDM was 20.6% and OGTT was performed to 66.3% of pregnant women [18]. Finally, our dataset was relatively small to be able to conclusively evaluate subgroups of chromosomal abnormalities, such as trisomies and sex chromosome abnormalities.

## Conclusion

Our study revealed a higher risk of major CAs, especially a higher risk of chromosomal abnormalities, in the offspring of GDM mothers compared to the non-diabetic controls. Given that GDM is typically diagnosed in the second trimester, it should be considered not as a direct risk factor for CAs or chromosomal abnormalities but rather as a signal of a longer-term metabolic imbalance of the mother, originating already before pregnancy.

## Data Availability

The data that support the findings are not publicly available because access to the registry data requires permission from the registry authorities. The register used in the study is maintained by the Finnish Institute for Health and Welfare. Researchers can apply similar register-data from Findata, Finnish Social and Health Data Permit Authority: https://findata.fi/en/.
